# Pharmacokinetic changes and therapeutic drug monitoring of lamotrigine during pregnancy

**DOI:** 10.1002/brb3.1315

**Published:** 2019-05-18

**Authors:** Ye Ding, Xiaoping Tan, Shuo Zhang, Yang Guo

**Affiliations:** ^1^ Department of Neurology Shengjing Hospital of China Medical University, Tiexi District Shenyang China

**Keywords:** epilepsy, lamotrigine, pharmacokinetics, pregnancy, therapeutic drug monitoring

## Abstract

**Objectives:**

To evaluate the pharmacokinetic changes in lamotrigine (LTG) from prepregnancy to postpartum and to assess the impact of therapeutic drug monitoring (TDM) on seizure management during pregnancy in a Chinese population.

**Methods:**

A series of women who were on LTG monotherapy before conception or during pregnancy were included in this retrospective study. The clinical characteristics of the mothers and fetuses were collected. The apparent clearance (AC) and the ratio to target concentration (RTC) were calculated for each trimester or for each month. RTCs were compared between patients with and without an increase in the frequency of seizures. A receiver operating characteristic curve to determine the RTC threshold, which predicts increased seizure frequency best, was drawn.

**Results:**

A total of 12 patients and their 12 pregnancies were reviewed retrospectively. AC increased by 82.5% during the first trimester (*p* = 0.0343), 203.2% during the second trimester (*p* = 0.0010), and 197.0% during the third trimester (*p* = 0.0061) compared with the prepregnancy level. The value returned to the prepregnancy level after delivery. Seven patients who had adequate baseline information were included to examine the association between serum LTG concentration and seizure frequency. The RTC values of patients with and without an increased frequency of seizures were significantly different (*p* = 0.0164), and increased seizure frequency was associated with a lower RTC. An RTC < 0.64 was a predictor of deteriorating seizures.

**Conclusions:**

The pharmacokinetic changes in LTG during pregnancy displayed marked interpatient variation. TDM can support a rational treatment plan for LTG use during pregnancy. We recommend regular monitoring of LTG serum concentrations from prepregnancy to postpartum.

## 1 INTRODUCTION

Epilepsy during pregnancy poses a special challenge to neurologists worldwide. The treatment must balance the risk of seizures with the potential for adverse effects from drug use which may affect both the mother and developing fetus (Bech et al., 2014; Cohen‐Israel et al., 2018; Vajda et al., 2017). Lamotrigine (LTG) is a second‐generation antiepileptic drug (AED) with broad spectrum activity. It is considered an ideal AED administered to women of childbearing age and during pregnancy (Yasam et al., 2016). Previous studies have reported that the pharmacokinetics of LTG are substantially affected by pregnancy (Fotopoulou et al., 2009; Milosheska et al., 2016; Pennell et al., 2004, 2008; Polepally et al., 2014; Reisinger, Newman, Loring, Pennell, & Meador, 2013), so a dose adjustment may be needed under therapeutic drug monitoring (TDM) during pregnancy. However, there is a lack of Chinese data until now, regardless of the sample size. The aims of the present study were to evaluate the pharmacokinetic changes in LTG from prepregnancy to postpartum and to assess the impact of TDM on seizure management during pregnancy in a Chinese population.

## 2 METHODS

### 2.1 Patient characteristics

Women who were pregnant or planning pregnancy and who suffered from epilepsy and were treated with LTG during January 2014–April 2018 at Shengjing Hospital, China Medical University were screened for inclusion in the present retrospective study. All patients met the criteria for the diagnosis of epilepsy issued by the International League against Epilepsy in 2017 (Scheffer et al., 2017) and received LTG monotherapy. Patients were excluded for age <17 years, cardiopulmonary dysfunction, renal or hepatic dysfunction, significant mental retardation (IQ <70), depression, schizophrenia, or other serious mental disorders, and coadministration of medications known to influence the pregnancy outcome. Patients on an AED polytherapy regimen that could contribute to seizure control were excluded. All subjects received no concomitant drugs interacting with LTG before or after pregnancy, including enzyme‐inducing antiepileptic drugs, estrogen‐containing contraceptives, or antipsychotic drug such as sertraline. This study was approved by the Ethics Committee for Clinical Trials of Shengjing Hospital (no. 2018PS417K).

Age of delivery, onset age of epilepsy, gestational age (GA) at birth, sex and birth weight of the fetus, seizure type, changes in the frequency of epileptic seizures during pregnancy compared with 1 year before pregnancy, LTG serum concentrations (before conception, during pregnancy, and postpartum), LTG dose, and health status of the fetus were collected. GA was determined based on last menstrual period. The LTG serum concentration was then classified as preconception, first trimester (≤13 weeks GA), second trimester (14–27 weeks GA), third trimester (≥28 weeks GA–delivery), or postpartum. Blood samples were taken before the morning dose, which was at least 6 hr after the last dose. The LTG dosage was adjusted according to the patients' seizure type, seizure frequency, and individual target concentrations. The target serum concentration for each patient is the ideal concentration at which seizures are well controlled without adverse effects (Pennell et al., 2008). Results of the LTG serum concentrations were actively used for TDM to maintain stable levels throughout pregnancy. The laboratory analysis was performed by high‐pressure liquid chromatography. As LTG presents only moderate protein binding capacity, we determined the total concentration values (Clark, Klein, Perel, Helsel, & Wisner, 2013; Fotopoulou et al., 2009; Pennell et al., 2008). The formula for calculating apparent clearance (AC) was: AC (mg/[mg/L]) = daily dose (mg)/serum concentration (mg/L) (Pennell et al., 2004). Prepregnancy target LTG serum concentrations and baseline seizure frequencies were collected from the patients to assess the association between LTG serum concentration and seizure frequency. We also calculated the ratio of LTG concentration to individual target concentration (RTC). These values were calculated for each trimester and for each month. A receiver operating characteristic (ROC) curve was drawn to determine the threshold of RTC and to predict the increased seizure frequency.

### 2.2 Statistical analyses

The statistical analyses (descriptive statistics, *t* test, chi‐square test, and ROC curve) were conducted with IBM SPSS Statistics Version 19.0 (IBM Corp., Armonk, NY). A *p* < 0.05 was considered significant.

## 3 RESULTS

A total of 12 patients and their 12 pregnancies were included (Table 1). The mean age of delivery was (28.6 ± 5.4) years (range, 20–41 years). No premature births or deformities were detected. Of the 12 patients, five (41.7%) had no seizures during pregnancy and seven (58.3%) had seizures. There was a significant difference in seizures between the first and the second trimester (*p* = 0.0304), but no significant difference between the second and the third trimester (*p* = 0.0917).

**Table 1:** Study population characteristics

Patient number	Pregnancy number	Age of delivery (year)	Age of epilepsy onset (year)	Gestational age at birth (week)	Dose used prior to pregnancy (mg)	Target concentration (mg/L)	Birth weight of fetus (g)	Sex of fetus	Apgar score of fetus	Seizure types before pregnancy	Types and numbers of seizures in		
											1st trimester	2nd^a^ trimester	3rd trimester
1	1	41	8	39	—	—	3,100	F	10	FIA	—	FIA1	—
2	2	30	16	38	150	2.73	3,300	F	10	GTC, FIA	FIA1	FIA3	—
3	3	27	16	38	—	—	3,600	M	10	GTC, FIA	—	FIA2	FIA3
4	4	22	14	38	100	2.72	3,400	M	10	GTC, FIA	—	GTC3, FIA1	—
5	5	26	12	38	50	1.80	2,700	F	10	GTC	—	—	—
6	6	30	10	38	—	—	2,850	F	10	GTC	—	—	—
7	7	30	1	39	75	1.43	3,000	M	10	GTC	—	GTC2	—
8	8	20	10	37	50	1.90	3,000	F	10	GTC, FIA	—	FIA2	—
9	9	33	14	39	100	3.18	3,400	F	10	GTC	—	—	—
10	10	28	10	37	—	—	3,050	M	10	GTC	—	—	—
11	11	26	18	39	—	—	3,300	F	10	GTC	—	—	—
12	12	30	24	40	125	2.33	3,570	M	10	GTC, FIA	—	GTC1	GTC1

Note

The number behind represents the number of seizure attacks.

Abbreviations: FIA, focal impaired awareness; GTC, generalized tonic–clonic seizure.

a χ^2^ test for seizures between the first and the second trimesters (*p* = 0.0304), χ^2^ test for seizures between the second and the third trimester (*p* = 0.0917).

**Figure 1:**
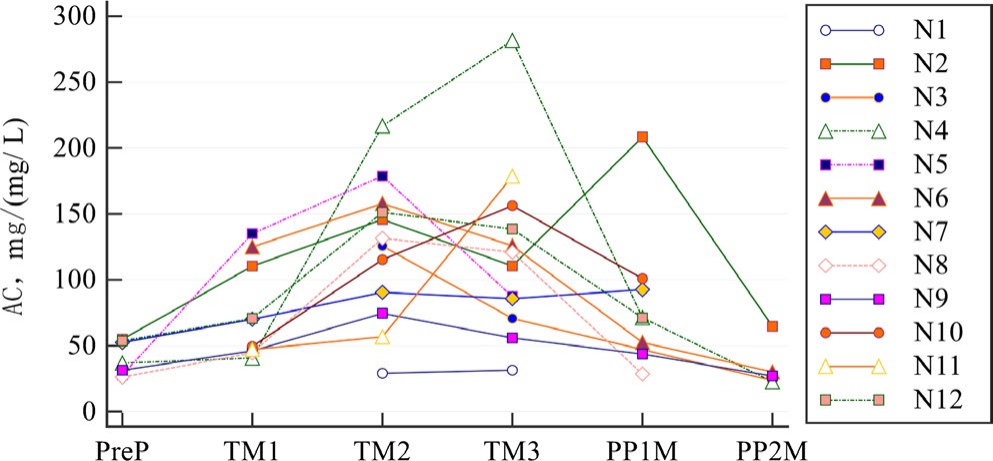
Individual changes in AC from prepregnancy to postpartum. AC, apparent clearance; N, patient number; TM1, TM2, TM3, first, second, and third trimester, respectively; PP1M, PP2M, first and second month postpartum, respectively; PreP, prepregnancy values

The AC of LTG across the pregnancy and postpartum periods showed individual differences (Figure 1). A large variation in AC values was observed among the patients. Compared with the prepregnancy level, AC increased by 82.5% during the first trimester (*p* = 0.0343), 203.2% during the second trimester (*p* = 0.0010), and by 197.0% during the third trimester (*p* = 0.0061). After delivery, the value returned to the prepregnancy level (Table 2).

**Table 2:** Mean AC values

Stages of pregnancy	Mean AC (mg/[mg/L])	Percentage changes^a^	*SD*	Number of patients	Number of samples	*p*‐value
Prepregnancy	40.5		12.8	7	7	
1st trimester	73.9	82.5	36.1	10	13	0.0343
2nd trimester	122.8	203.2	53.2	12	25	0.0010
3rd trimester	120.3	197.0	66.0	12	28	0.0061
PP1M	83.6	106.4	55.9	8	8	0.0683
PP2M	33.6	−17.0	17.6	5	5	0.4495

Abbreviations: AC, apparent clearance; PP1M, PP2M, first and second month postpartum, respectively; *SD*, standard deviation.

a Percentage change compared with values before pregnancy.

Seven patients (nos. 2, 4, 5, 7, 8, 9, and 12), who had adequate baseline information about prepregnancy target serum concentrations and seizure frequencies, were included to examine the association between LTG serum concentration and seizure frequency (Table 3).

**Table 3:** Percentages of patients with increased seizure frequency compared with the prepregnancy baseline by gestational age and months postpartum

Month	1	2	3	4	5	6	7	8	9	10	PP1	PP2
Patients (%)	0	0	14.3	28.6	57.1	14.3	28.6	0	14.3	0	0	0

**Figure 2:**
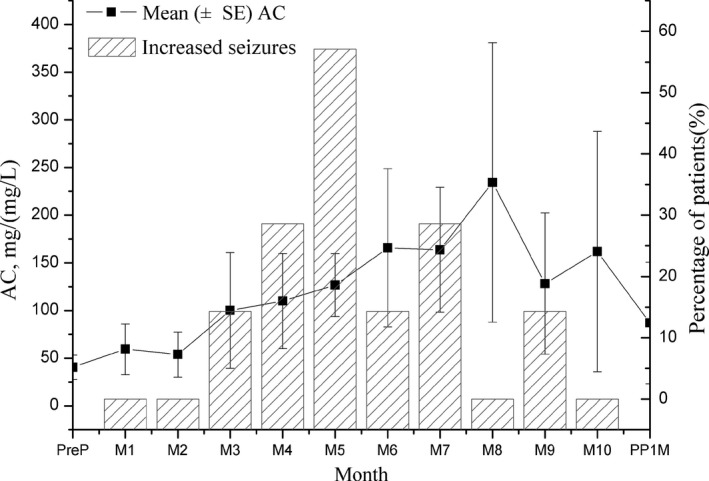
The AC of LTG and increased seizures during pregnancy and postpartum period. Mean (±*SE*) AC and percentage of patients with increased seizure frequency compared with prepregnancy baseline, by month gestational age and month postpartum. AC, apparent clearance; LTG, lamotrigine; M, month

The percentage of patients who had an increase in seizures is plotted in Figure 2, along with the AC by month. Increased seizure frequency occurred in the highest percentage of patients during the fifth month of pregnancy (57.1%).

**Figure 3:**
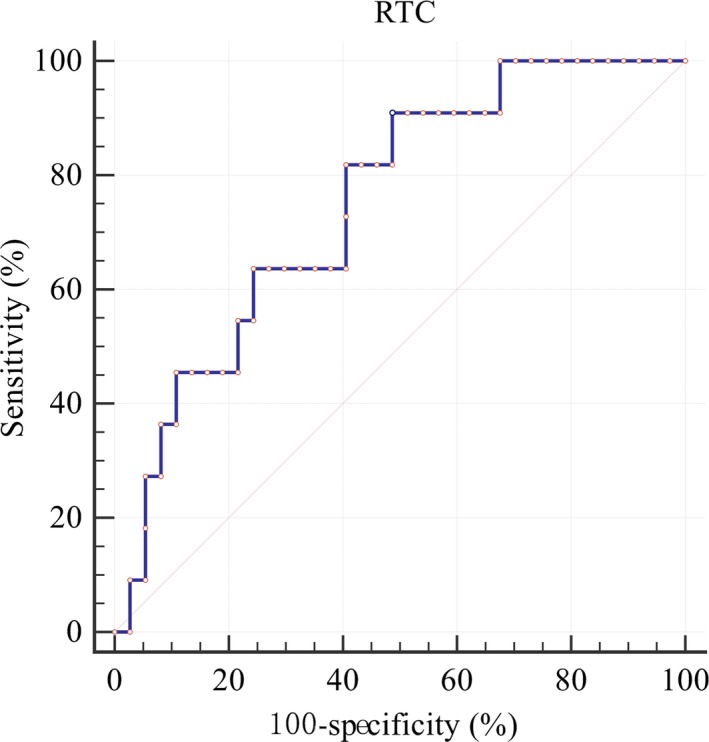
ROC curve of the RTC threshold for predicting increased seizure frequency. AUC = 0.749, 95% CI: (0.603–0.863), *p*‐value: 0.0013. AUC, area under the curve; CI, confidence interval; ROC, receiver operating characteristic; RTC, ratio of LTG concentration to target concentration

Forty‐eight RTC values were calculated. We performed the *t* test to compare the RTC values of patients who had an increased seizure frequency with the RTC values of those who did not. The RTC values of these two groups were significantly different (*p* = 0.0164), and increased seizure frequency was associated with a lower RTC. We drew the ROC curve for predicting increased seizure frequency with reference to Pennell et al. (2008), and calculated the RTC threshold that best predicted an increase of seizure frequency (Figure 3). A reduction in the RTC below the threshold was viewed as increased susceptibility to seizures. When the RTC threshold = 64.34%, a true‐positive rate of 90.91% and a true‐negative rate of 51.35% were achieved.

## 4 DISCUSSION

Lamotrigine is considered an ideal AED for pregnant women because of its effectiveness in treating epilepsy and the low risk of fetal malformations and altered cognitive development. LTG exposure carries the lowest risk for overall malformations based on current evidence (Weston et al., 2016). Exposure to LTG does not have detrimental effect on child neurodevelopment (Cummings, Stewart, Stevenson, Morrow, & Nelson, 2011). Longer term studies find no significant effect of lamotrigine on IQ at age 6 years (Baker et al., 2015; Cohen‐Israel et al., 2018). No premature births or deformities were detected in this study. Long‐term follow‐up is required to assess cognitive impairment.

Pregnancy influences the pharmacokinetics of LTG. This study confirmed previous reports of significant increases in clearance between preconception baseline and all trimesters of pregnancy. Whether there is a difference in LTG clearance change across racial groups is inconclusive. In a retrospective analysis of 95 pregnant women with epilepsy of which 69 pregnancies were controlled by LTG, Reisinger et al., (2013) found no difference between Caucasian, African American, and Asian patients in clearance change for all drugs combined or for LTG monotherapy. In another prospective study of 53 women treated with LTG monotherapy, Pennell et al found a difference in free LTG clearance between white and black patients, with the white patients exhibiting a higher clearance. But this trend was not observed for total LTG clearance (Pennell et al., 2008). In this study, the extent of change in clearance was in the middle of the range compared with previous studies (Fotopoulou et al., 2009; Milosheska et al., 2016; Pennell et al., 2004, 2008; Polepally et al., 2014; Reisinger et al., 2013). In a larger US‐based cohort study, the majority (77%) of women had a substantial increase in the oral clearance by the end of pregnancy, whereas 23% of women had a minimal increase (Polepally et al., 2014). We could not look for this because of our small sample size. But when we view Figure 1, it does appear that our cohort could also fall into different subpopulations with N4 in the high clearance change group and N9 in the low clearance change group.

At present, TDM may not be fully and correctly utilized. Some studies indicate that LTG dose changes were often not guided by TDM (Richards, Reith, Stitely, & Smith, 2018). The effectiveness of TDM and serum LTG during pregnancy for improving seizure control has been demonstrated (Fotopoulou et al., 2009; de Haan et al., 2004; Pennell et al., 2008; Petrenaite, Sabers, & Hansen‐Schwartz, 2005; Pirie et al., 2014; Sabers, 2012). No uniform standard exists on how to use TDM during pregnancy. Also, there are no specific guidelines for monitoring frequency and duration of pregnancy. Some adjustments have been based entirely on serum concentrations (Sabers, 2012), while others had reference to clinical manifestations (Pennell et al., 2008). The frequency of monitoring mentioned in previous reports was from once every 3 months (Petrenaite et al., 2005) to once every 1–3 months (Pennell et al., 2008) to once every 4 weeks (Fotopoulou et al., 2009; Sabers, 2012). The monitoring frequency of this study was once every 1–3 months. Recent studies show that increases in LTG clearance can begin as early as 5 weeks GA, often before women know they are pregnant (Karanam et al., 2018). Many doctors and patients in China have not paid sufficient attention to TDM during pregnancy, leading to missing baseline or early concentrations. Our study confirms that planning of pregnancy is necessary and we should employ TDM as early as possible even before pregnancy and in the first month of GA. Specific data such as maternal weight, GA, LTG dose, time interval since the last dose, seizure types, seizure frequencies, and side effects should be recorded at every visit.

A reduction in RTC led to worsening seizures in this study. An RTC < 0.64 was a predictor of deteriorating seizures. A dosage adjustment is necessary for women with epilepsy on LTG monotherapy during pregnancy when the LTG serum concentration is <64% of the target concentration. Coincidentally, the threshold is close to previous reports (Pennell et al., 2008; Reisinger et al., 2013).

Some limitations of this study include partial loss of prepregnancy data, such as some weight and baseline serum concentrations. Additionally, we did not include weights in the clearance calculations, because patient weights were not available at some time points. However, AC also makes sense so that some studies adopted this variable (Pennell et al., 2004). In addition to AC, some studies used a ratio of serum concentration to daily dose (serum concentration/daily dose, [mg/L]/mg) to measure the changes in pharmacokinetics (de Haan et al., 2004; Petrenaite et al., 2005), regardless of body weight. Correspondingly, the ratio decreased during pregnancy compared with that before pregnancy. The study is also limited by the small sample size. We need to expand the sample size for further research. However, even these small numbers represent an addition to the Chinese data literature.

## 5 CONCLUSIONS

The pharmacokinetic changes in LTG during pregnancy displayed marked interpatient variation in this retrospective study. TDM can support a rational treatment plan for LTG use during pregnancy. We recommend regular monitoring of LTG serum concentrations from prepregnancy to postpartum. As TDM for pregnancy starts relatively late in China, both doctors and patients should pay more attention. Improving patient compliance is indispensable. Further effort is needed to develop dose adjustment regimes and treatment paradigms.

## CONFLICTS OF INTEREST

The authors declare no conflicts of interest in this study.

## Data Availability

The data that support the findings of this study are available on request from the corresponding author. The data are not publicly available due to privacy or ethical restrictions.

## References

[brb31315-bib-0001] Baker, G. A. , Bromley, R. L. , Briggs, M. , Cheyne, C. P. , Cohen, M. J. , Garcia‐Finana, M. , … Clayton‐Smith, J. (2015). IQ at 6 years after in utero exposure to antiepileptic drugs: A controlled cohort study. Neurology, 84(4), 382–390. 10.1212/wnl.0000000000001182 25540307 PMC4336006

[brb31315-bib-0002] Bech, B. H. , Kjaersgaard, M. I. , Pedersen, H. S. , Howards, P. P. , Sorensen, M. J. , Olsen, J. , … Christensen, J. (2014). Use of antiepileptic drugs during pregnancy and risk of spontaneous abortion and stillbirth: Population based cohort study. BMJ, 349, g5159 10.1136/bmj.g5159 25150301 PMC4141333

[brb31315-bib-0003] Clark, C. T. , Klein, A. M. , Perel, J. M. , Helsel, J. , & Wisner, K. L. (2013). Lamotrigine dosing for pregnant patients with bipolar disorder. American Journal of Psychiatry, 170(11), 1240–1247. 10.1176/appi.ajp.2013.13010006 24185239 PMC4154145

[brb31315-bib-0004] Cohen‐Israel, M. , Berger, I. , Martonovich, E. Y. , Klinger, G. , Stahl, B. , & Linder, N. (2018). Short‐ and long‐term complications of in utero exposure to lamotrigine. British Journal of Clinical Pharmacology, 84(1), 189–194. 10.1111/bcp.13437 29044597 PMC5736833

[brb31315-bib-0005] Cummings, C. , Stewart, M. , Stevenson, M. , Morrow, J. , & Nelson, J. (2011). Neurodevelopment of children exposed in utero to lamotrigine, sodium valproate and carbamazepine. Archives of Disease in Childhood, 96(7), 643–647. 10.1136/adc.2009.176990 21415043

[brb31315-bib-0006] de Haan, G. J. , Edelbroek, P. , Segers, J. , Engelsman, M. , Lindhout, D. , Devile‐Notschaele, M. , & Augustijn, P. (2004). Gestation‐induced changes in lamotrigine pharmacokinetics: A monotherapy study. Neurology, 63(3), 571–573. 10.1212/01.WNL.0000133213.10244.FD 15304599

[brb31315-bib-0007] Fotopoulou, C. , Kretz, R. , Bauer, S. , Schefold, J. C. , Schmitz, B. , Dudenhausen, J. W. , & Henrich, W. (2009). Prospectively assessed changes in lamotrigine‐concentration in women with epilepsy during pregnancy, lactation and the neonatal period. Epilepsy Research, 85(1), 60–64. 10.1016/j.eplepsyres.2009.02.011 19272754

[brb31315-bib-0008] Karanam, A. , Pennell, P. B. , French, J. A. , Harden, C. L. , Allien, S. , Lau, C. , … Birnbaum, A. K. (2018). Lamotrigine clearance increases by 5 weeks gestational age: Relationship to estradiol concentrations and gestational age. Annals of Neurology, 84(4), 556–563. 10.1002/ana.25321 30168175

[brb31315-bib-0009] Milosheska, D. , Lorber, B. , Vovk, T. , Kastelic, M. , Dolzan, V. , & Grabnar, I. (2016). Pharmacokinetics of lamotrigine and its metabolite N‐2‐glucuronide: Influence of polymorphism of UDP‐glucuronosyltransferases and drug transporters. British Journal of Clinical Pharmacology, 82(2), 399–411. 10.1111/bcp.12984 27096250 PMC4972156

[brb31315-bib-0010] Pennell, P. B. , Newport, D. J. , Stowe, Z. N. , Helmers, S. L. , Montgomery, J. Q. , & Henry, T. R. (2004). The impact of pregnancy and childbirth on the metabolism of lamotrigine. Neurology, 62(2), 292–295. 10.1212/01.WNL.0000103286.47129.F8 14745072

[brb31315-bib-0011] Pennell, P. B. , Peng, L. , Newport, D. J. , Ritchie, J. C. , Koganti, A. , Holley, D. K. , … Stowe, Z. N. (2008). Lamotrigine in pregnancy: Clearance, therapeutic drug monitoring, and seizure frequency. Neurology, 70(22 Pt 2), 2130–2136. 10.1212/01.wnl.0000289511.20864.2a 18046009 PMC3589527

[brb31315-bib-0012] Petrenaite, V. , Sabers, A. , & Hansen‐Schwartz, J. (2005). Individual changes in lamotrigine plasma concentrations during pregnancy. Epilepsy Research, 65(3), 185–188. 10.1016/j.eplepsyres.2005.06.004 16084694

[brb31315-bib-0013] Pirie, D. A. J. , Wattar, B. H. A. , Pirie, A. M. , Houston, V. , Siddiqua, A. , Doug, M. , … Thangaratinam, S. (2014). Effects of monitoring strategies on seizures in pregnant women on lamotrigine: A meta‐analysis. European Journal of Obstetrics, Gynecology, and Reproductive Biology, 172, 26–31. 10.1016/j.ejogrb.2013.10.021 24211103

[brb31315-bib-0014] Polepally, A. R. , Pennell, P. B. , Brundage, R. C. , Stowe, Z. N. , Newport, D. J. , Viguera, A. C. , … Birnbaum, A. K. (2014). Model‐based lamotrigine clearance changes during pregnancy: Clinical implication. Annals of Clinical and Translational Neurology, 1(2), 99–106. 10.1002/acn3.29 24883336 PMC4038031

[brb31315-bib-0015] Reisinger, T. L. , Newman, M. , Loring, D. W. , Pennell, P. B. , & Meador, K. J. (2013). Antiepileptic drug clearance and seizure frequency during pregnancy in women with epilepsy. Epilepsy & Behavior, 29(1), 13–18. 10.1016/j.yebeh.2013.06.026 23911354 PMC3775962

[brb31315-bib-0016] Richards, N. , Reith, D. , Stitely, M. , & Smith, A. (2018). Are doses of lamotrigine or levetiracetam adjusted during pregnancy? Epilepsia Open, 3(1), 86–90. 10.1002/epi4.12086 29588992 PMC5839303

[brb31315-bib-0017] Sabers, A. (2012). Algorithm for lamotrigine dose adjustment before, during, and after pregnancy. Acta Neurologica Scandinavica, 126(1), e1–4. 10.1111/j.1600-0404.2011.01627.x 22150770

[brb31315-bib-0018] Scheffer, I. E. , Berkovic, S. , Capovilla, G. , Connolly, M. B. , French, J. , Guilhoto, L. , … Zuberi, S. M. (2017). ILAE classification of the epilepsies: Position paper of the ILAE Commission for Classification and Terminology. Epilepsia, 58(4), 512–521. 10.1111/epi.13709 28276062 PMC5386840

[brb31315-bib-0019] Vajda, F. J. , O'Brien, T. J. , Graham, J. E. , Hitchcock, A. A. , Lander, C. M. , & Eadie, M. J. (2017). Antiepileptic drugs, foetal malformations and spontaneous abortions. Acta Neurologica Scandinavica, 135(3), 360–365. 10.1111/ane.12672 27573510

[brb31315-bib-0020] Weston, J. , Bromley, R. , Jackson, C. F. , Adab, N. , Clayton‐Smith, J. , Greenhalgh, J. , … Marson, A. G. (2016). Monotherapy treatment of epilepsy in pregnancy: Congenital malformation outcomes in the child. Cochrane Database Systematic Review, 11, CD010224 10.1002/14651858.CD010224.pub2 PMC6465055 27819746

[brb31315-bib-0021] Yasam, V. R. , Jakki, S. L. , Senthil, V. , Eswaramoorthy, M. , Shanmuganathan, S. , Arjunan, K. , & Nanjan, M. J. (2016). A pharmacological overview of lamotrigine for the treatment of epilepsy. Expert Review of Clinical Pharmacology, 9(12), 1533–1546. 10.1080/17512433.2016.1254041 27825017

